# Authors’ correction for Euro Surveill. 2012;17(22)

**DOI:** 10.2807/1560-7917.ES.2019.24.27.1907041

**Published:** 2019-07-04

**Authors:** Eurosurveillance editorial team

**Affiliations:** 1 European Centre for Disease Prevention and Control (ECDC), Stockholm, Sweden

In the article ‘Mandatory and recommended vaccination in the EU, Iceland and Norway: results of the VENICE 2010 survey on the ways of implementing national vaccination programmes’ by M Haverkate, et al. published on 31 May 2012, the modalities of implementation of the childhood vaccination programme for Latvia were corrected in Tables 1 and 2. The affected numbers in the text were also corrected accordingly. These corrections were made on 3 July 2019, upon request of the authors.

